# Exploratory biomarkers for oxaliplatin-induced nivolumab responsiveness in metastatic microsatellite-stable colorectal cancer

**DOI:** 10.1038/s41416-026-03357-6

**Published:** 2026-02-23

**Authors:** Anne Hansen Ree, Paula A. Bousquet, Tina Visnovska, Torben Lüders, Benjamin P. Geisler, Shixiong Wang, Diana L. Bordin, Hilde L. Nilsen, Hanne M. Hamre, Christian Kersten, Eva Hofsli, Marianne G. Guren, Halfdan Sorbye, Jens P. Berg, Kjersti Flatmark, Sebastian Meltzer

**Affiliations:** 1 Department of Oncology, Akershus University Hospital, Lørenskog, Norway; 2 Institute of Clinical Medicine, University of Oslo, Oslo, Norway; 3 Department of Clinical Molecular Biology, Akershus University Hospital, Lørenskog, Norway; 4 Institute of Health and Society, University of Oslo, Oslo, Norway; 5 Department of Research, Sørlandet Hospital, Kristiansand, Norway; 6 Department of Oncology, St. Olav’s Hospital, Trondheim, Norway; 7 Department of Clinical and Molecular Medicine, Norwegian University of Science and Technology, Trondheim, Norway; 8 Department of Oncology, Oslo University Hospital, Oslo, Norway; 9 Cancer Clinic, Haukeland University Hospital, Bergen, Norway; 10 Department of Clinical Science, University of Bergen, Bergen, Norway; 11 Department of Medical Biochemistry, Oslo University Hospital, Oslo, Norway; 12 Department of Surgical Oncology, Oslo University Hospital, Oslo, Norway; 13 Department of Tumour Biology, Oslo University Hospital, Oslo, Norway

**Keywords:** Colorectal cancer, Colorectal cancer

## Abstract

**Background:**

The randomised METIMMOX trial evaluated short-course oxaliplatin-based chemotherapy alternating with nivolumab for metastatic microsatellite-stable/mismatch repair-proficient colorectal cancer. In a *post hoc* analysis, we investigated whether tumour mutations or patients’ systemic inflammation might provide insights into responsiveness to the METIMMOX regimen.

**Methods:**

Patients received either oxaliplatin-based chemotherapy (control group) or alternating two cycles each of chemotherapy and nivolumab (experimental group), with progression-free survival (PFS) as the primary endpoint. Tumour biopsies were sequenced with the TruSight Oncology 500 assay.

**Results:**

The median tumour mutational burden (TMB; in mutations/megabase) was 8 (range, 1–13). The experimental-arm patients with TMB ≥9 or *BRAF*-V600E mutation (*n* = 17) achieved median PFS of 19.8 months (95% confidence interval, 11.3–28.3), longer (*p* = 0.0090) than experimental-arm patients with TMB < 9 not *BRAF*-V600E (*n* = 19) and control-arm patients with either TMB and *BRAF* status combination (*n* = 31). With TMB ≥9 or *BRAF*-V600E and normal, non-inflammatory level of C-reactive protein when starting nivolumab (*n* = 11), median PFS was 35.0 months (95% confidence interval, 6.8–63.0; *p* < 0.0001).

**Conclusions:**

TMB, somatic *BRAF* status and systemic inflammation should be prospectively investigated as practical biomarkers for predicting potential responsiveness to immune checkpoint inhibitors in metastatic microsatellite-stable/mismatch repair-proficient colorectal cancer.

## Background

Immune checkpoint inhibitors (ICIs) have significantly improved the outcome of local, locally advanced and metastatic colorectal cancer (CRC) for the subgroup of patients with the highly immunogenic microsatellite-instable (MSI)/mismatch repair (MMR)-deficient entity [1–4]. Additionally, rare patient subgroups with ICI responsiveness have been reported [5–7]. However, most CRC cases are microsatellite-stable (MSS)/MMR-proficient with inherently low immunogenicity [8], devoid of innate ICI responsiveness [9]. Unresectable abdominal metastases from MSS/MMR-proficient CRC commonly reflect an aggressive phenotype [10] and have been considered ICI-resistant [11, 12].

The randomised METIMMOX trial evaluated the combination of short-course oxaliplatin-based chemotherapy alternating with nivolumab for previously untreated, unresectable abdominal metastases from MSS/MMR-proficient CRC [13]. A subgroup of patients assigned to this experimental schedule had remarkably extended progression-free survival (PFS)—the primary endpoint—compared to the control-group patients given the chemotherapy only [13, 14], while treatment resistance among other study subjects was a concern [13]. Thus, with the introduction of combined-modality treatments that include ICIs, pragmatic biomarkers of efficacy or early failure are highly advisable [15].

In CRC, the tumour mutational burden (TMB) is a proxy for the tumour neoantigen burden [16], and the ICI-responsive MSI/MMR-deficient CRC entity presents median TMB of 55 mutations/megabase [17]. The measure of TMB ≥ 10 was in 2020 reported to be associated with ICI responsiveness in metastatic disease from several tumour types; the study, however, excluded CRC [18]. Nevertheless, this data accelerated the pan-cancer approval in the USA of single-agent pembrolizumab for TMB ≥ 10 cases, a decision that was rapidly criticised [6]. For CRC in particular, not all of mutations relating to TMB ≥ 10 enhance ICI responsiveness [19].

Among the MSS/MMR-proficient CRC cases, patients with tumours with the activating *BRAF*-V600E mutation, comprising 14% (across stages) of a Scandinavian population-based cohort [20], have particularly poor prognosis for metastatic disease with median overall survival of a year or less in pooled data analysis [21]. However, the randomised BREAKWATER study recently showed that the combination of oxaliplatin-based chemotherapy with inhibitors directly targeting the active tumour signalling pathways as first-line therapy improves outcome significantly [22]. In the METIMMOX experimental arm of alternating short-course oxaliplatin-based chemotherapy and nivolumab, patients with tumour harbouring the *BRAF*-V600E driver mutation experienced complete response (CR) and notably long-lasting PFS [14].

Inflammation of the tumour microenvironment (TME) is typical in CRC [23, 24]. The host systemic inflammatory response has considerable prognostic value in localised CRC [25]. Moreover, systemic inflammation is present in many patients with advanced CRC [26] and impairs ICI efficacy in MSI/MMR-deficient disease [27]. For the METIMMOX experimental-arm patients, a C-reactive protein (CRP) level within the reference limit was associated with nivolumab response [13]. In other ICI trials, alternative measures of systemic inflammation have demonstrated superior prognostic value compared to tumour-based factors. A low value of the modified Glasgow Prognostic Score (mGPS), which categorises risk groups based on CRP and albumin values, identified patients who might benefit from continuing ICI treatment beyond the time of radiologic progressive disease [28]. A high neutrophil-to-lymphocyte ratio (NLR) is a poor prognostic factor in metastatic CRC, and baseline NLR < 3 was associated with improved survival outcomes in patients with chemotherapy-refractory metastatic CRC receiving an experimental ICI regimen [29].

In the present *post hoc* analysis, we explored how tumour mutations, primarily in terms of TMB or *BRAF*-V600E, or patients’ systemic inflammation might have selected the patients with exceptional response to the METIMMOX regimen.

## Methods

### Study design, participants and endpoints

The details of the METIMMOX trial have been described previously [13]. Briefly, eligible patients had previously untreated, unresectable abdominal (liver, peritoneal, nodal) metastases from MSS/MMR-proficient CRC. Tumour *RAS/BRAF* mutational status was determined according to routine clinical procedures. The MSS/MMR-proficient status was determined by the absence of tumour microsatellite instability markers (by PCR analysis; one of two routine assays was accepted) or the presence of MMR proteins (MLH1, MSH2, MSH4/6, PMS2; by immunohistochemistry), conducted by accredited molecular pathology laboratories. To be included in the study, the result of only one test was required, but the MSS/MMR-proficient status was confirmed for all subjects by both immunohistochemistry and PCR analysis (and both PCR assays for those who achieved CR) for the *post hoc* analyses. The 80 study patients were randomly assigned to treatment with the FLOX regimen (oxaliplatin 85 mg/m^2^ day 1 and 5-fluorouracil 500 mg/m^2^ and folinic acid 100 mg days 1–2) Q2W (control arm) or alternating 2 cycles each of FLOX Q2W and nivolumab (240 mg) Q2W (experimental arm), with prespecified break periods (Supplementary Fig. S1). The serum CRP level was measured (as routine clinical analysis with reference limit <5.0 mg/L) at every study visit, as were other routine blood tests. Radiologic response was assessed every 8 weeks by blinded independent central review with PFS as the primary endpoint.

### TMB assessment and variant analysis

Next-generation sequencing (NGS) was done using the TruSight Oncology 500 DNA/RNA Assay and is detailed in Supplementary information. Functional annotation was performed using the Personal Cancer Genome Reporter software package [30]. TMB (in mutations/megabase, reported as integers) was based on coding non-synonymous single-nucleotide variants and insertions/deletions with a variant-allele frequency of ≥5% with the effective panel size in megabases as the denominator. The somatic mutation data were visualised in the Maftools R package [31].

### Statistical methods

PFS was expressed as median with 95% confidence interval (CI) based on Kaplan–Meier estimates, and differences were assessed with the log-rank test. Cox regression models were estimated to determine potential associations between PFS and circulating inflammatory factors. Group differences were assessed by the Mann–Whitney U test, Kruskal–Wallis test or Fisher’s exact test, as appropriate. All tests were two-sided. Analyses were conducted using SPSS v29 or GraphPad Prism v10.

## Results

### Patient and tumour characteristics

Patients were enrolled between 29 May 2018 and 22 October 2021. End of study was 18 March 2024. Subject assignment (CONSORT diagram: Supplementary Fig. S2) to the control arm (*n* = 40, with 38 initiating treatment) and experimental arm (*n* = 40, with 38 initiating treatment) has been detailed previously [13]. Since in the experimental arm, two patients left the study after the first FLOX cycle and moreover, the first two therapy cycles were identical across both study arms (halfway towards the first radiologic reassessment), the population of the current analysis comprised all subjects who adhered to the treatment until this formal reassessment, which enabled objective comparison of the study arms. Sequencing data were acquired from this population (31 control-arm patients and 36 experimental-arm patients; NGS population). Baseline characteristics, along with tumour mutations identified through routine clinical practice, are presented in Table 1.

**Table 1:** Baseline patient and tumour characteristics.

			All (*n* = 67)		Experimental arm (*n* = 36)		Control arm (*n* = 31)	
			TMB < 9 (*n* = 44)	TMB ≥9 (*n* = 23)	TMB < 9 (*n* = 21)	TMB ≥9 (*n* = 15)	TMB < 9 (*n* = 23)	TMB ≥9 (*n* = 8)
Median age, years (range)			64 (41–80)	65 (38–77)	60 (43–80)	60 (48–72)	65 (41–79)	65 (38–77)
Sex	Female		26 (59)	12 (52)	10 (48)	7 (47)	8 (35)	5 (63)
	Male		18 (41)	11 (48)	11 (52)	8 (53)	15 (65)	3 (37)
ECOG performance status	0		21 (48)	15 (65)	12 (57)	10 (67)	11 (48)	5 (63)
	1		23 (52)	8 (35)	9 (43)	5 (33)	12 (52)	3 (37)
Primary tumour site	Right colon		12 (27)	7 (30)	4 (19)	6 (40)	8 (35)	1 (12)
	Left colon		17 (39)	12 (52)	12 (57)	5 (33)	5 (22)	7 (88)
	Rectum		15 (34)	4 (17)	5 (24)	4 (27)	10 (43)	0 (0)
Number of metastatic sites	1		11 (25)	6 (26)	5 (24)	3 (20)	6 (26)	3 (37)
	≥2		33 (75)	17 (74)	16 (76)	12 (80)	17 (74)	5 (63)
Involved liver	No		6 (14)	5 (22)	2 (10)	5 (33)	4 (17)	0 (0)
	Yes		38 (86)	18 (78)	19 (90)	10 (67)	19 (83)	8 (100)
*KRAS* status^a^	Wildtype		22 (50)	10 (43)	10 (48)	5 (33)	12 (52)	5 (63)
	A146T/V		1 (2)	2 (9)	0 (0)	2 (13)	1 (4)	0 (0)
	G12A, G12C, G12V, G13A		9 (20)	7 (30)	4 (19)	5 (33)	5 (22)	2 (25)
	G12D, G12R, G12S, G13D		9 (20)	3 (13)	6 (29)	3 (20)	3 (13)	0 (0)
	Q61L/H, K117X		3 (7)	1 (4)	1 (5)	0 (0)	2 (9)	1 (13)
*BRAF* status	Wildtype		37 (84)	21 (91)	19 (90)	14 (93)	18 (78)	7 (88)
	V600E		7 (16)	2 (9)	2 (10)	1 (7)	5 (22)	1 (12)

*ECOG* Eastern Cooperative Oncology Group, *TMB* tumour mutational burden (in mutations/megabase).

^a^No *NRAS*-mutant cases in the population of the current analysis.

For the experimental-arm cases, median TMB was 8 (range, 1–13); for the control-arm cases, median TMB was 7 (range, 2–13). The TMB distribution was comparable for right-sided (*n* = 19), left-sided (*n* = 29) and rectal (*n* = 19) primary tumours (Supplementary Fig. S3).

### TMB and *BRAF* status for PFS prediction

The results presented here are based on data as of the date of study conclusion. The primary endpoint had not been reached at 33–40 months for three experimental-arm patients. One patient had ongoing stable disease with the *MLH1*-K618A mutation and the two others ongoing CR as the best overall radiologic response. None exhibited somatic *POLE/POLD1* mutations (Supplementary Table S1).

**Fig. 1:**
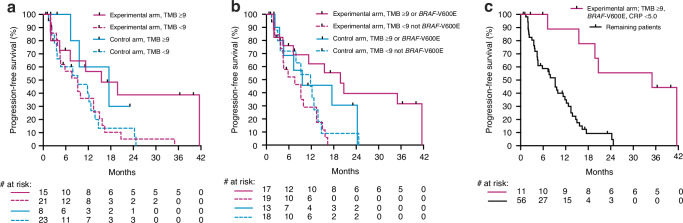
Kaplan–Meier curves of progression-free survival. The 67 subjects were stratified by **a** study arm and tumour mutational burden (TMB), **b** study arm, TMB and tumour *BRAF* status or **c** experimental-arm assignment and the combination of TMB ≥9 or *BRAF*-V600E and C-reactive protein (CRP) within the reference limit when starting nivolumab *versus* all other combinations of strata.

Median PFS was 9.9 months (range, 1.9–41.6) for experimental-arm patients and 11.8 months (range, 0.7–24.7) for control-arm cases, both longer than the values previously reported for the intention-to-treat population [13] due to the exclusion of patients who did not reach the first post-baseline reassessment in the current analysis. Given the emphasis on evaluating responses to the experimental-arm therapy, the median TMB cutoff of 8 (≥9 *versus* < 9 by integers) was used for stratification. As illustrated in Fig. 1a, the experimental-arm patients with TMB ≥9 (*n* = 15) reached median PFS 15.5 months (95% CI, 2.5–28.4). In comparison (*p* = 0.039), the experimental-arm patients with TMB < 9 (*n* = 21) had median PFS 9.2 months (95% CI, 4.4–13.9), control-arm patients with TMB ≥9 (*n* = 8) had median PFS 17.5 months (95% CI, 5.2–29.8) and control-arm patients with TMB < 9 (*n* = 23) had median PFS 9.2 months (95% CI, 0.5–17.9).

We reported previously that the experimental-arm patients with tumour harbouring the commonly poor-prognostic *BRAF*-V600E driver mutation experienced early disappearance of the primary tumour followed by CR of all overt metastatic disease, resulting in unexpectedly long-lasting PFS [14]. Combining the TMB and *BRAF* mutational status (Fig. 1b), the experimental-arm patients with TMB ≥9 or *BRAF*-V600E (*n* = 17) reached median PFS 19.8 months (95% CI, 11.3–28.3). In comparison (*p* = 0.0090), experimental-arm patients with TMB < 9 not *BRAF*-V600E (*n* = 19) had median PFS 7.6 months (95% CI, 1.5–13.6), control-arm patients with TMB ≥9 or *BRAF*-V600E (*n* = 13) had median PFS 9.6 months (95% CI, 0.0–22.1) and control-arm patients with TMB < 9 not *BRAF*-V600E (*n* = 18) had median PFS 11.8 months (95% CI, 5.6–18.0).

### An additional impact of systemic inflammation

We showed previously that the response to the experimental METIMMOX regimen might reflect the ability of the initial two chemotherapy cycles to repress tumour-induced systemic inflammation [13]. In the NGS population (Supplementary Fig. S4), the CRP measures (lacking for one patient at each of the study visits) declined over the initial two chemotherapy cycles (*p* = 0.043) from median 12.0 mg/L (range, 0.7–109; *n* = 66) at start of the first cycle to median 6.0 mg/L (range, 0.5–60.0; *n* = 66) at start of the third therapy cycle, from when the control-arm and experimental-arm patients proceeded with different regimens. Experimental-arm patients with TMB ≥9 or *BRAF*-V600E and CRP < 5.0 mg/L when starting nivolumab (*n* = 11) attained median PFS 35.0 months (95% CI, 6.8–63.0; *p* < 0.0001 *versus* all other combinations of strata with median PFS 9.2 months (95% CI, 6.9–11.5); Fig. 1c). Supplementary Fig. S5 outlines the distribution of patients with the various combinations of strata.

Moreover, in the experimental arm, patients with right-sided primary tumour had longer PFS than those with left-sided or rectal tumour (*p* = 0.041; Supplementary Fig. S6A). This might have been accounted for by the right-sided cases with CRP < 5.0 mg/L at the first nivolumab administration, who had longer PFS compared to those with CRP above the reference limit (*p* = 0.0011 by pairwise comparison; Supplementary Fig. S6B), suggesting an interaction between oxaliplatin-responsive systemic inflammation and the primary tumour site. Stratification by tumour site and TMB status together did not result in any PFS difference among experimental-arm patients (Supplementary Fig. S6C). For control-arm patients, PFS was comparable regardless of tumour site, TMB status and the CRP level after the initial two chemotherapy cycles (Supplementary Fig. S6D–F).

Given that a CRP level within the reference limit appeared permissive for a response to nivolumab, we sought to isolate the impact of changing CRP levels during the initial chemotherapy. An elevated CRP level alone at the start of nivolumab was associated with a significantly heightened progression risk (hazard ratio >7.5; Supplementary Table S2). However, neither the mGPS score nor NLR measured at baseline correlated with PFS in the experimental arm (Supplementary Table S3), contrasting with the clear prognostic value observed in trials utilising ICI without chemotherapy [28, 29].

### Mutations associated with TMB

**Fig. 2:**
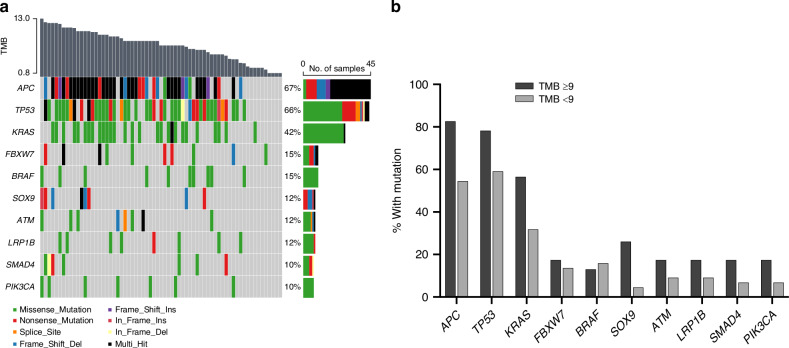
Genomic characterisation of the primary tumours. **a** Oncoplot visualising the most frequent gene alterations; each column represents one individual patient with colours indicating the mutation characteristics and co-occurring tumour mutational burden (TMB). **b** The 10 most frequently mutated genes within the TMB strata.

The mutational landscape of the primary tumour specimens was derived from the targeted exon sequencing data. As outlined for the entire NGS population (Supplementary Fig. S7), the median variant number was 10 (range, 1–19), most variants being missense mutations and C > T transitions. The 10 most frequently altered genes were found in 31 (86%) of the experimental-arm cases and 27 (87%) of the control-arm cases and were typical for CRC [32]. As expected for randomly assigned patients, the mutant genes had comparable frequencies in the study arms. The individual combinations of variants, together with the co-occurring TMB, are detailed in the Fig. 2a oncoplot.

In general, the 10 most frequently mutated genes were more prevalent in TMB ≥9 tumours, of which mutant *APC* (*p* = 0.032) and *SOX9* (*p* = 0.016) were significantly associated with the TMB status (Fig. 2b). Additionally, *KRAS* mutations were associated with co-occurring *APC* mutations (*p* < 0.001). The NGS analysis revealed that 10 (15%) of cases carried a *BRAF* mutation (8 V600E and 2 K601N; Fig. 2a, b). Of note, one of the *BRAF*-V600E cases identified through the routine clinical practice (Table 1), with measurable plasma *BRAF*-V600E at baseline [14], was not retrieved by the NGS analysis.

Three experimental-arm cases (TMB 10–12, PFS 5.9 and 15.5 months and one censored without disease progression at 40 months) and one control-arm case (TMB 7) harboured mutation in an MMR gene. One experimental-arm case (TMB 8, *POLE-*K1169fs, PFS 9.2 months) and two control-arm cases (TMB 8–10) held a *POLE/POLD1* mutation that, if causing proofreading deficiency and typically TMB > 100, is associated with ICI responsiveness [7]. For the four experimental-arm patients (only two with radiologic response), best overall response, PFS and all identified somatic mutations are detailed in Supplementary Table S4.

## Discussion

The METIMMOX population consisted of patients with newly diagnosed unresectable metastatic MSS/MMR-proficient CRC. To be eligible, patients were mandated to present with infradiaphragmatic (liver, peritoneal, nodal) metastases. After the study was launched in 2018, reports indicated that abdominal metastases from MSS/MMR-proficient CRC may be ICI resistant [11, 12]. Moreover, the METIMMOX patients were given a total of only eight cycles before treatment break (Supplementary Fig. S1), due to prevailing clinical routine and historical practice for chemotherapy-only patients [33]. For the experimental-arm patients this implied only four chemotherapy cycles over four months before a break. As a natural consequence, the METIMMOX go-and-stop schedule with de-intensified chemotherapy within a relatively brief treatment sequence was highly tolerable, even for the oldest patients aged 80 [13].

Despite these two premises, a subgroup of the experimental-arm subjects experienced remarkably extended PFS. The combination of TMB ≥9 or the somatic *BRAF*-V600E mutation and repressed systemic inflammation (CRP < 5.0 mg/L) after the initial two chemotherapy cycles resulted in the most superior outcome—median PFS almost four times longer than the median PFS for the entire intention-to-treat population [13].

In metastatic MSS/MMR-proficient CRC, intermediate-to-high TMB is found in a certain percentage of cases [19, 32, 34]. Large-scale genomic analysis has revealed discrepancy between the microsatellite-stability status and TMB in a range of malignancies; however, CRC was among the two entities showing strongest association between high TMB and MSI [35]. The 2020 study that reported ICI responsiveness in metastatic TMB ≥ 10 cancers excluded CRC [18]. A recent report on a range of advanced-stage tumour types confirmed that in MSS/MMR-proficient cohorts—except CRC—TMB ≥ 10 was associated with ICI benefit [36]. Studies on chemotherapy-refractory MSS/MMR-proficient CRC with TMB above 9–10 as cutoff value have not been conclusive as to whether single-agent ICI is efficacious [37, 38]. Moreover, TMB > 10 may be an independent factor for improved response to first-line standard chemotherapy and overall survival in metastatic MSS/MMR-proficient colon cancer [39]. For the METIMMOX control-arm patients TMB status was not associated with PFS.

The combination of ICI with chemotherapy in first-line treatment of metastatic MSS/MMR-proficient CRC has in other randomised trials, by *post hoc* analyses, identified responding patient subgroups characterised by TMB ≥ 10 or high-density immune-cell infiltration of the tumour (the AtezoTRIBE trial) or particular molecular subgroups (the CheckMate 9 × 8 trial) [40, 41]. Here, the experimental regimens consisted of ICI and chemotherapy given concomitantly. Different from this, the experimental METIMMOX regimen consisted of alternating short-course chemotherapy and ICI. The concept was built on our previous findings for short-course oxaliplatin-containing chemotherapy in locally advanced or early metastatic CRC, indicating that oxaliplatin may invoke tumour-defeating immunity [42–44]. Specifically, patients who presented unresectable single-organ liver metastases as the first metastatic event, given oxaliplatin as hepatic arterial infusion chemotherapy and responding with a rapid rise in a circulating anti-tumour immune factor, were alive 8–12 years later [44].

In our analyses of the data from the intention-to-treat population [13], we noticed that the METIMMOX regimen resulted in CR with PFS as long as 21–42 months (still ongoing for two subjects at end of study) in cases essentially characterised by right-sided primary tumour, female sex, age older than 60 and intermediate TMB or the somatic *BRAF*-V600E mutation. It has long been known that sporadic (non-heritable) MSI CRC is associated with concomitant *BRAF*-V600E (presenting a distinct immune-permissive feature [45] and high lymph node yield [46]), right-sided primary tumour, female sex and increasing age [46–48]. As these retrospective findings were reported long before ICI was used to treat metastatic MSI/MMR-deficient CRC, overall survival to standard chemotherapies was found to be inferior compared to MSS cases [47, 48]. Recent whole-genome sequencing of >2,000 CRC patient samples revealed that right-sided MSS CRC without high TMB resembles MSI CRC [16]. A decade ago, preclinical findings indicated that the combined inhibition of *BRAF*-activated and MAPK signalling pathways augmented the tumour immune response [49]. Recent trial data from metastatic MSS/MMR-proficient *BRAF*-V600E CRC have shown the induction of tumour-intrinsic immune signatures in patients responding to this combination of signalling pathway inhibitors [50] and moreover, that the further addition of nivolumab caused radiologic response in patients who exhibited tumour MAPK signalling and immune activation signatures [51]. The breadth of these data provides a rationale for therapeutic strategies that can invoke ICI responsiveness in MSS/MMR-proficient CRC with the concurrent *BRAF*-V600E mutation.

The single-arm MEDITREME trial for patients with metastatic MSS/MMR-proficient and *RAS*-mutant CRC administered first-line therapy as dual-ICI concomitantly with oxaliplatin-based chemotherapy for six cycles over three months before the chemotherapy was stopped and maintenance single-ICI continued until progression [52]. Despite dissimilarities, the MEDITREME regimen consisted of a limited number of chemotherapy cycles like the METIMMOX regimen. Integrated transcriptomic analysis of MEDITREME tumour specimens, corroborated with immunohistochemistry, showed that signatures enriched in immune-related pathways were associated with exceptional response. These findings also comprised patients with liver metastases, another analogy with the METIMMOX trial. T-cell receptor sequencing of resected liver metastases from a MEDITREME patient with partial response showed TME accumulation of a cluster of polyfunctional cytotoxic T-cell clones, resembling our own observation of clonal T-cell expansion in liver metastases using the same approach [14]. These findings support the notion that de-intensified oxaliplatin-containing chemotherapy can convert a de novo ICI-resistant intrahepatic TME to ICI responsiveness. Of note, the ongoing POCHI trial of first-line oxaliplatin-based chemotherapy and bevacizumab in combination with pembrolizumab enrols metastatic MSS/MMR-proficient CRC patients presenting with a high de novo level of cytotoxic T-cells (in the primary tumour). The initial data presented in October 2024 indicated approximately half of patients with involved liver and a fifth with CR [53].

Besides intermediate TMB and *BRAF*-V600E, no particular somatic mutations (except the one case exhibiting the *MLH1*-K618A variant that has no or mild impairment of the protein function) prevailed in the patients with exceptional responses to the METIMMOX regimen. The absence of systemic inflammation, however, seemed to be a decisive determinant of oxaliplatin-induced nivolumab responsiveness. Approximately 30% of all CRC patients present with systemic inflammation [54], which alone is associated with treatment resistance and impaired disease-specific and overall survival [55]. CRC is regarded the archetype malignancy in which inflammation suppresses TME immune responses and may outperform the mutational landscape with regard to outcome [23].

The findings presented here came from *post hoc* analyses not prespecified in the study protocol and thus without the required statistical power, weakening their validity. Importantly, we did not correct for testing of multiple subgroups, and potential interaction terms were not formally assessed. Another weakness derives from the determination of panel-based TMB scoring, which is not yet universally standardised [56, 57] with regard to factors such as panel size, gene content and the applied bioinformatics pipeline [58], thus complicating comparisons across studies.

In conclusion, biomarkers such as TMB, the somatic *BRAF*-V600E mutation and repressed systemic inflammation after the initial chemotherapy may be associated with extended PFS for patients with abdominal metastases from MSS/MMR-proficient CRC given first-line alternating oxaliplatin-based chemotherapy and nivolumab. The concept of selecting a subgroup of patient with metastatic MSS/MMR-proficient CRC for ICI therapy using such biomarkers will be prospectively investigated, with sufficient statistical power, in a follow-up METIMMOX study.

## Supplementary information

Supplementary information

## Data Availability

Requests for data can be made to the corresponding author. The data generated in this study are subject to patient confidentiality in accordance with the General Data Protection Regulation of the European Union, and the transfer of data or materials will require approval from the Data Privacy Officer at Akershus University Hospital and on some occasions from the Regional Committee for Medical and Health Research Ethics of South-East Norway. Any shared data will be de-identified.
