# Supplementary material for: Exploratory biomarkers for oxaliplatin-induced nivolumab responsiveness in metastatic microsatellite-stable colorectal cancer
Source: Br J Cancer. 2026 Feb 23;134(8):1176–82. doi: 10.1038/s41416-026-03357-6 (PMC13036003; doi:10.1038/s41416-026-03357-6)
Supplement: Supplementary file 1 — Supplementary information [file 41416_2026_3357_MOESM1_ESM.pdf]

SUPPLEMENTARY INFORMATION

DNA/RNA sequencing

For DNA/RNA extraction, formalin-fixed paraffin-embedded primary tumour specimens were processed using the AllPrep DNA/RNA FFPE Mini Kit (Qiagen) or Mag-Bind FFPE DNA/RNA 96 Kit (Omega Bio-Tek). Concentrations of DNA and RNA were quantified using a Qubit fluorometer (Thermo Fisher Scientific), and quality assessment was conducted by the Infinium FFPE QC Kit (Illumina) for DNA and the TapeStation HS RNA Kit (Agilent) for RNA. Analysis by next-generation sequencing was performed by the TruSight Oncology 500 DNA/RNA Assay (Illumina), achieving targeted exon coverage of 600-1850× for DNA samples. Data analysis was done in the TruSight Oncology 500 Local App version 2.2 Software (Illumina), followed by an in-house post-processing pipeline, which included manual quality checks to eliminate sequencing artefacts and germline variants.

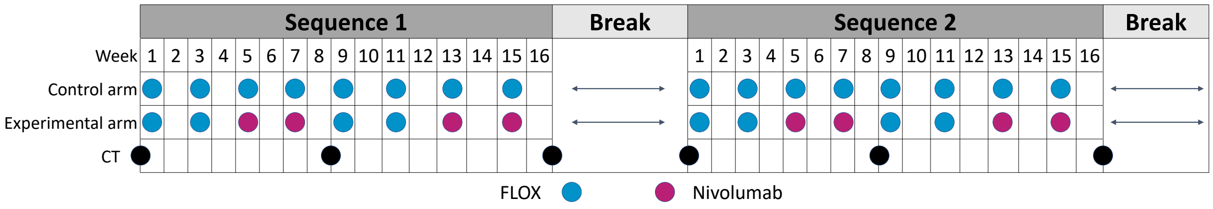

Supplementary Fig. S1 The METIMMOX trial schedule.

Coloured circles indicate study visits. The treatment schedule consisted of intermittent periods of eight cycles. FLOX: oxaliplatin 85 mg/m^2 day 1 and bolus 5-fluorouracil 500 mg/m^2 and folinic acid 100 mg days 1-2; intravenous administration Q2W. Nivolumab: 240 mg flat dose; intravenous administration Q2W. During a break period, radiographic assessment (CT) and visits were done every 8 weeks until disease progression and the treatment was reintroduced in a new sequence. Treatment sequences were continued until disease progression on ongoing therapy (progressive disease), an intolerable adverse event, consent withdrawal or death, whichever occurred first.

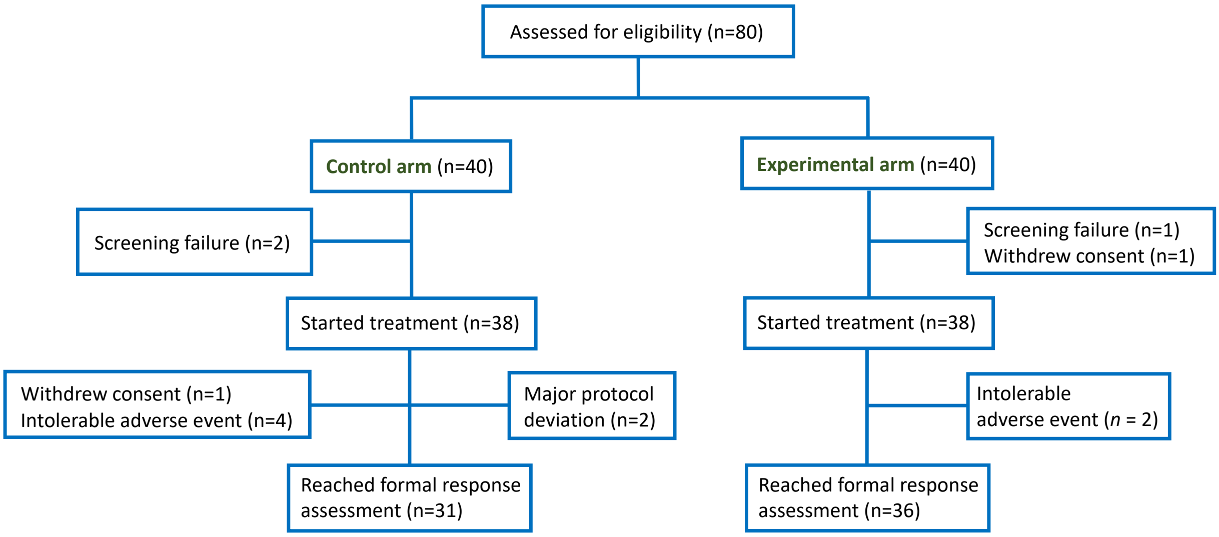

Supplementary Fig. S2 CONSORT diagram of the METIMMOX subjects.

The trial enrolled 40 control-arm patients to FLOX and 40 experimental-arm patients to alternating two cycles each of FLOX and nivolumab. Four cases were screening failures or withdrew the informed consent before the first FLOX cycle was administered, leaving 76 intention-to-treat patients randomly allocated between the study arms. Seven control-arm patients left the study before the first post-baseline radiographic assessment, resulting in 31 per-protocol cases. In the experimental arm, two patients left the study after the first FLOX cycle, resulting in 36 per-protocol cases.

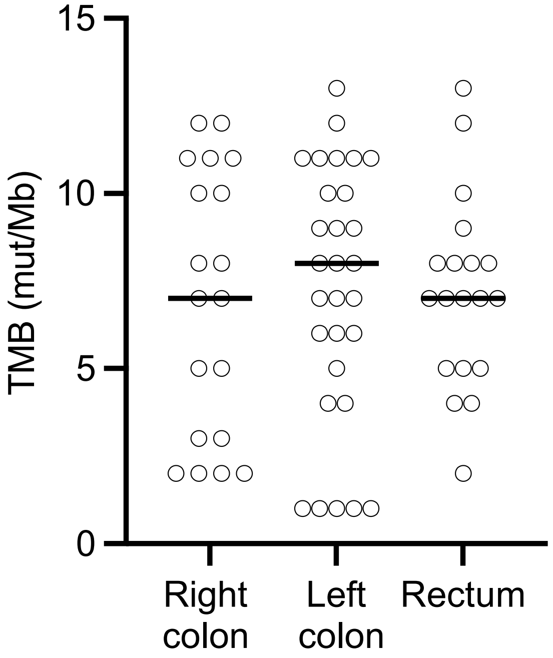

Supplementary Fig. S3 Distribution of the tumour mutational burden (TMB) with regard to the primary tumour site (right colon, *n* = 19; left colon, *n* = 29; rectum, *n* = 19). Horizontal lines: Median values.

Mb megabase, *mut* mutations.

Supplementary Table S1. Patient and tumour characteristics for the subjects who had not reached the primary endpoint at the date of study conclusion.

Sex	Age (years)	Primary tumour site	Somatic mutations	TMB	BOR	Censoring (months)
Male	58	Right colon	*MLH1*-K618A, *FGF19*-A14V, *HIST1H3G*-K65R, *IKZF1*-E401K, *INPP4A*-R544H, *KDR*-A1166T, *NOTCH1*-G1001S, *PIK3CA*-P449T, *PIK3C3*-A855G, *PPARG*-I167M, *RNF43*-R132X, *SLIT2*-T1021I, *SMAD4*-A532_L533delinsV, *STAG1*-E1214K, *TP53*-C238Y	12	SD	40.0
Female	72	Right colon	*KRAS*-G12V, *ALK*-G740R, *APC*-R216X, *APC*-Q1291X, *CDKN2C*-V130A, *DOT1L*-T444M, *FGF3*-R181H, *GATA4*-R43W, *MGA*-G1124R, *MGA*-I2864V, *NF1*-K1036I, *PARK2*-H303N, *PDGFRA*-P60T, *PTPN11*-S326C, *TBX3*-G598X, *TP53*-E271Q, *TP53*-E224K, *TP53*-G262S	11	CR	36.4
Female	69	Right colon	*BRAF*-V600E, *APC*-T1556fs, *AR*-S697P, *ATRX*-K994fs, *BCOR*-R1699Q, *ERBB4*-M1154T, *FANCA*-E420K, *FGF14*-D101G, *LRP1B*-G3981R, *LZTR1*-T428M, *MDC1*-E407K, *NFKBIA*-R218Q, *PTPRT*-V179M, *STAT3*-NA, *TP53*-R282W, *TSC2*-R585C	12	CR	33.1

BOR best overall response, *CR* complete response, *SD* stable disease, *TMB* tumour mutational burden (in mutations/megabase).

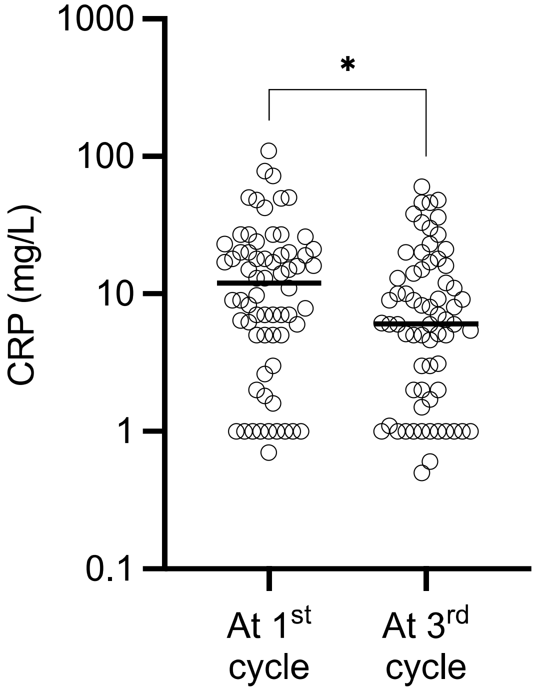

Supplementary Fig. S4 C-reactive protein (CRP).

Circulating levels at start of the first (*n* = 66) and third (*n* = 66) therapy cycles, lacking for one patient at each of the study visits. Horizontal lines: Median values.

* $p < 0.05$.

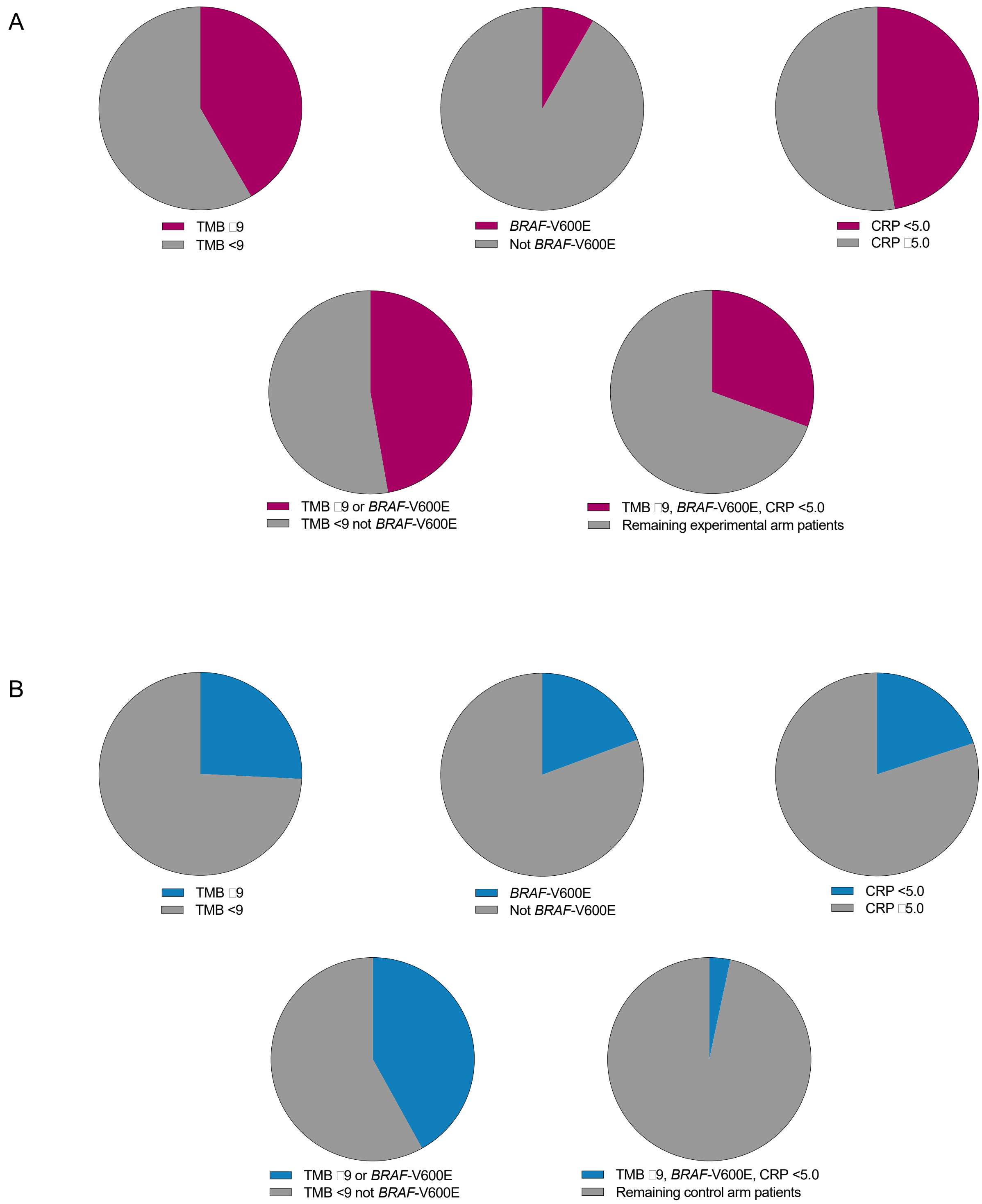

Supplementary Fig. S5 Distribution of patients with various combinations of strata in the experimental arm (A) and control arm (B).

CRP C-reactive protein (level in mg/L) at start of the third therapy cycle, *TMB* tumour mutational burden (in mutations/megabase).

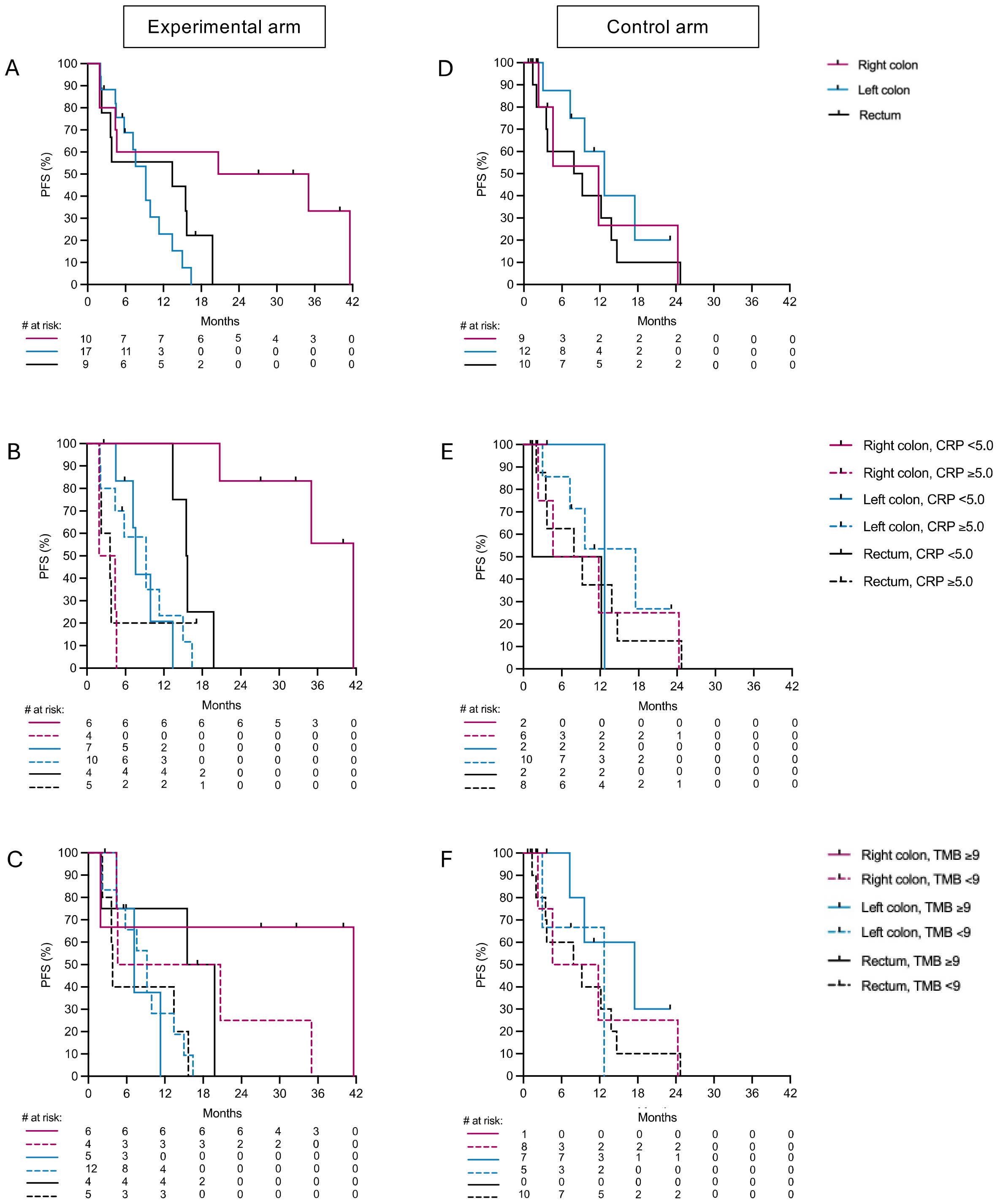

Supplementary Fig. S6 Kaplan-Meier curves of progression-free survival (PFS).

The 67 subjects were stratified by the study arm, primary tumour site (right colon, left colon or rectum), level of C-reactive protein (CRP; in mg/L) at start of the third therapy cycle and tumour mutational burden (TMB; in mutations/megabase), as indicated in panels A-F.

Supplementary Table S2. Potential associations between change of the level of C-reactive protein (CRP) from the start of the first to the third therapy cycles and progression-free survival, by Cox regression.

**CRP at 1st cycle →**	**Experimental arm**			**Control arm**		
**CRP at 3rd cycle**	***n***	**HR (95% CI)**	***p***	***n***	**HR (95% CI)**	***p***
within ref → within ref	7	1		4	1	
above ref → within ref	10	2.46 (0.65-9.33)	0.19	3	0.57 (0.06-5.57)	0.63
within ref → above ref	1	14.3 (1.31-157)	0.029	4	1.20 (0.19-7.65)	0.85
above ref → above ref	18	7.51 (2.01-28.1)	0.003	19	0.72 (0.20-2.66)	0.63

CI confidence interval, *HR* hazard ratio, *ref* reference limit (<5.0 mg/L).
The CRP profile is missing for one control-arm patient.

Supplementary Table S3. Potential associations between baseline systemic inflammatory factors and progression-free survival, by Cox regression.

	**Experimental arm**			**Control arm**		
	n	**HR (95% CI)**	***p***	***n***	**HR (95% CI)**	***p***
NLR	36	1.14 (0.95-1.37)	0.15	31	0.96 (0.75-1.23)	0.75
mGPS 0	18	1		15	1	
mGPS 1	18	1.60 (0.76-3.38)	0.22	15	1.03 (0.39-2.72)	0.95
mGPS 2	0			1	2.72 (0.32-23.5)	0.36

CI confidence interval, *HR* hazard ratio, *mGPS* modified Glasgow Prognostic Score, *NLR* neutrophil-to-lymphocyte ratio.
The mGPS categorises risk groups based on the C-reactive protein and albumin values.

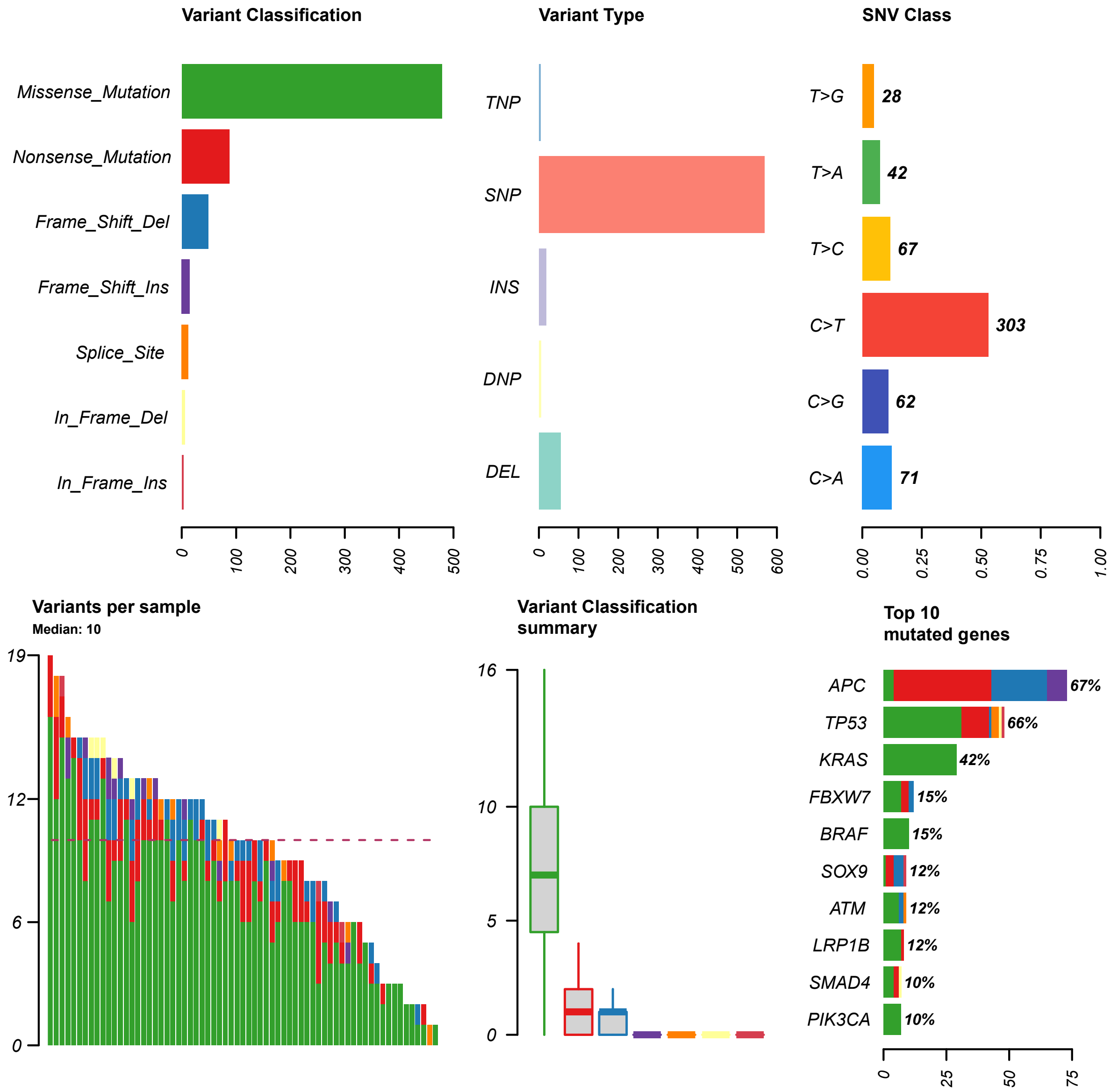

Supplementary Fig. S7 Summary of the somatic mutational profiles.

Top left: Distribution of different mutation classifications. Top middle: Frequencies of the mutation types. Top right: Proportion of different single-nucleotide variant (SNV) substitutions across the patient samples. Bottom left: The number of mutations per patient sample, categorised according to the classification; dashed line: the median variant number. Bottom middle: The distribution of different functional mutation types across the patient samples. Bottom right: The most frequently mutated genes.

Supplementary Table S4. Patient and tumour characteristics for the experimental-arm cases with somatic mutations in an MMR gene or *POLE/POLD1*.

Sex	Age (years)	Primary tumour site	Somatic mutations	TMB	BOR	PFS (months)
Male	58	Right colon	*MLH1*-K618A, *FGF19*-A14V, *HIST1H3G*-K65R, *IKZF1*-E401K, *INPP4A*-R544H, *KDR*-A1166T, *NOTCH1*-G1001S, *PIK3CA*-P449T, *PIK3C3*-A855G, *PPARG*-I167M, *RNF43*-R132X, *SLIT2*-T1021I, *SMAD4*-A532_L533delinsV, *STAG1*-E1214K, *TP53*-C238Y	12	SD	40.0 (censored without reaching the primary endpoint)
Male	72	Rectum	*MSH2*-N835D, *KRAS*-A146T, *APC*-E1284fs, *APC*-R1158fs, *AXIN2*-R841Q, *CEBPA*-P42S, *EPHA5*-K745E, *ERBB4*-R106H, *FBXW7*-K374N, *PARP1*-I706V, *RANBP2*-K99Q, *TP53*-Y234H	10	PR	15.5
Female	63	Left colon	*MSH2*-L362S, *APC*-A1492fs, *APC*-R876X, *BAP1*-R150H, *BTK*-R490C, *FLT4*-R275Q, *JAK3*-A174P, *LZTR1*-R340X, *MED12*-R1854H, *PTPRT*-V122I, *ROS1*-I1788M, *SOX9*-K137fs, *SOX9*-K137_T138delinsNL, *TP53*-R213X	11	SD	5.9
Male	57	Left colon	*POLE*-K1169fs, *KRAS*-G12S, *APC*-L1488fs, *ATM*-NA, *JAK3*-C591Y, *KDM5-A*Y472C, *PIK3R1*-K423X, *RICTOR*-K921X, *TP53*-R248Q, *ZFHX3*-T1379M	8	PR	9.2

BOR best overall response, *PFS* progression-free survival, *PR* partial response, *SD* stable disease, *TMB* tumour mutational burden (in mutations/megabase).
